# The Evolution of Sinus Tarsi Syndrome—What Is the Underlying Pathology?—A Critical Review

**DOI:** 10.3390/jcm12216878

**Published:** 2023-10-31

**Authors:** Madeleine Willegger, Maryse Bouchard, Gilbert M. Schwarz, Lena Hirtler, Andrea Veljkovic

**Affiliations:** 1 Department of Orthopedics and Trauma Surgery, Division of Orthopedics, Medical University of Vienna, Währinger Gürtel 18-20, 1090 Vienna, Austria; gilbert.schwarz@meduniwien.ac.at; 2 Department of Orthopaedics, Faculty of Medicine, University of British Columbia, St. Paul’s Hospital, Vancouver, BC V6T 1Z4, Canada; docveljkovic@yahoo.com; 3 Department of Surgery, Division of Orthopaedics, University of Toronto, Hospital for Sick Children, Toronto, ON M5S 1A1, Canada; maryse.bouchard@sickkids.ca; 4 Center for Anatomy and Cell Biology, Division of Anatomy, Medical University of Vienna, Währinger Straße 13, 1090 Vienna, Austria

**Keywords:** sinus tarsi syndrome, sinus tarsi pain, sinus tarsi impingement, tarsal sinus, subtalar joint, subtalar joint complex, subtalar instability, hindfoot instability, ankle instability

## Abstract

**Background and Objectives:** Sinus tarsi syndrome (STS) is defined as pain located at the lateral opening of the tarsal sinus. The exact etiology of sinus tarsi syndrome is not completely understood. Some do not believe it to be a true pathology. This review aims to clarify the definition of sinus tarsi syndrome to better understand the underlying pathologies. We further propose an algorithm to evaluate sinus tarsi pain and provide advice for consecutive treatment options. **Design**: This is a narrative review. By searching PubMed, the available current literature was reviewed. Articles were critically analyzed to determine the pathoanatomy, biomechanics, and etiology of sinus tarsi pain. Algorithms for clinical evaluation, diagnosis, and treatment were also recorded. Finally, the authors approach to evaluating and treating sinus tarsi pain was included in this review. **Results**: Reviewing the available literature, STS seems to be a catch-all phrase used to describe any pain in this anatomic region. Many causes of sinus tarsi pain were listed, including impingement, subtalar instability, and many other pathologies around the ankle. **Conclusions**: A thorough evaluation of patients presenting with pain in the sinus tarsi or instability of the hindfoot is essential to determining the underlying cause. When the cause of pain is still not clear after clinical exam and radiologic assessment, subtalar arthroscopy can be helpful as both a diagnostic and treatment tool. We propose that the term of STS should be avoided and that a more accurate diagnosis be used when possible. Once a diagnosis is made, appropriate treatment can be initiated.

## 1. Introduction

Sinus tarsi syndrome (STS) is defined as pain around the lateral opening of the tarsal sinus and may include symptoms of hindfoot instability. This definition is broad and non-specific, and current literature fails to elucidate a clear cause of STS. Some authors have even questioned the existence of STS as a true anatomical pathology [1].

The first description of STS was by O’Connor in 1958 [2]. He considered it a nonspecific pain located laterally at the tarsal sinus region as a consequence of previous trauma [2]. STS has since been reported in athletes such as dancers, basketball players, volleyball players, and patients with obesity and flatfoot deformity [2,3,4]. None of these papers, however, include a clear etiology or mechanism causing STS or provide diagnostic criteria, clinical or radiographic [2,4,5]. STS is therefore a clinical diagnosis made based on the patient’s medical history and clinical examination. Some authors report hindfoot instability in patients with STS; however, other reports specifically contradict the association [6,7,8].

The treatment of STS ranges from non-operative modalities, such as sinus tarsi injections with local anesthetics and corticosteroids, to surgical procedures including denervation, open and arthroscopic debridement of the sinus tarsi, and stabilization of the subtalar joint complex [1,4,7,9,10,11,12,13].

Nevertheless, STS remains a topic of controversy. The condition is poorly defined, with no diagnostic criteria or clear treatment algorithm. Sinus tarsi pain may be a symptom of a wide range of underlying foot and ankle pathologies. This review aims to examine the current literature on sinus tarsi syndrome. We summarize the tarsal sinus anatomy, biomechanics of the subtalar joint complex, possible etiologies and differential diagnoses, the clinical work-up, and treatment options.

## 2. Methods

In order to better assess the debate of STS and the etiology of sinus tarsi pain, a review of the available literature was performed in September 2022 using the PubMed database, searching the following keywords: “sinus tarsi syndrome”, “sinus tarsi pain”, “sinus tarsi impingement”, “sinus tarsi anatomy”, “subtalar joint”, “subtalar joint anatomy”, “subtalar biomechanics”, “subtalar instability” and “subtalar arthroscopy”. Titles and abstracts were screened for relevance by two independent reviewers.

PRISMA guidelines were not applicable, as the design of this review was not defined as a systematic review but as a critical review. The primary aim was not only to identify articles that addressed sinus tarsi syndrome but also to screen for manuscripts that studied related topics. Full-text articles were retrieved, and their references were further searched for additional relevant studies and book chapters to supplement the electronic database searches. The available literature was summarized, and evidence on STS, including the anatomy, biomechanics, etiology, clinical evaluation, diagnosis, and treatment, was critically reviewed. The authors added an overview of the underlying anatomy, as well as their personal experience diagnosing and treating sinus tarsi pain, including illustrative cases.

## 3. Results

### 3.1. Anatomy

**Figure 1:**
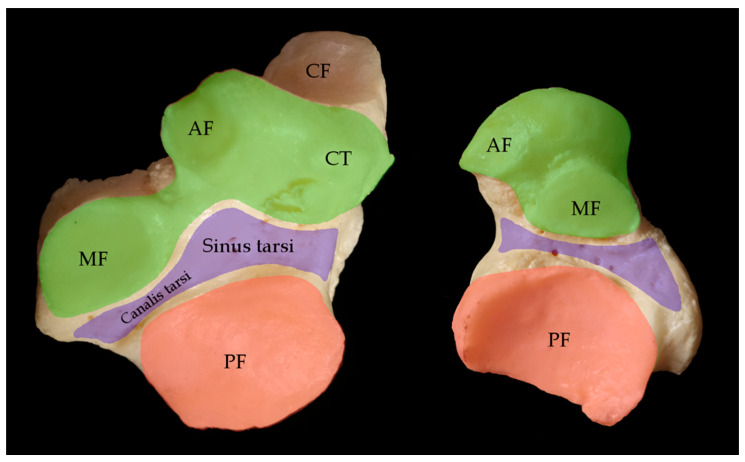
**Compartments of the subtalar joint complex**. Articular facets of the subtalar and calcaneonavicular joint complex of a right foot. Dorsal view of the calcaneus on the left, plantar view of the talus on the right site. The anterior compartment (green) consists of the anterior facet (AF), the middle facet (MF) and the crista lateralis (CT). The middle compartment (violet) is a conical tunnel, formed by the tarsal canal and tarsal sinus. The posterior compartment (red) consists of the posterior facet (PF). CF = cuboid facet.

The talotarsal joint is subdivided into the subtalar joint (or talocalcaneal joint) and the talocalcaneonavicular joint. The border between these joints is the tarsal sinus, or sinus tarsi. Morphologically, the talotarsal region has been described as being composed of three compartments: Anterior, middle, and posterior (Figure 1). The anterior compartment consists of the talocalcaneonavicular joint and is characterized as a ball-and-socket joint. In it, the talar head is supported by the anterior and middle articulating facets of the calcaneus and the spring or plantar calcaneonavicular ligament, forming the so-called acetabulum pedis with the posterior surface of the navicular bone [7,14,15,16]. The middle compartment is a conical interosseous tunnel, formed by the concave parts of the talus and calcaneus, consisting of the narrow posteromedial tarsal canal and wider anterolateral tarsal sinus that separate the talocalcaneonavicular joint from the subtalar joint (Figure 1). The long axis of the tarsal canal and tarsal sinus is obliquely oriented at an angle of 45° from posteromedial to anterolateral. The dimensions of the canal are 10 to 15 mm high, 3 to 5 mm wide, and 15 to 20 mm long [17].

The tarsal canal and tarsal sinus contain a complex system of ligaments, arteries and their anastomoses, adipose tissue, joint capsules, and nerve fibers [18]. The artery of the tarsal sinus is formed by the anastomoses of various arteries originating from the lateral aspect of the foot, primarily including branches from the proximal lateral tarsal artery and the lateral malleolar artery. This artery of the sinus tarsi may also be described as a vascular sling, as it anastomoses with the artery of the tarsal canal. The latter originates from the tibial artery and is responsible for the major blood supply of the talar body [18,19]. The tarsal canal and sinus tarsi are innervated by the tibial nerve, as well as the deep and superficial peroneal nerves [15].

**Figure 2:**
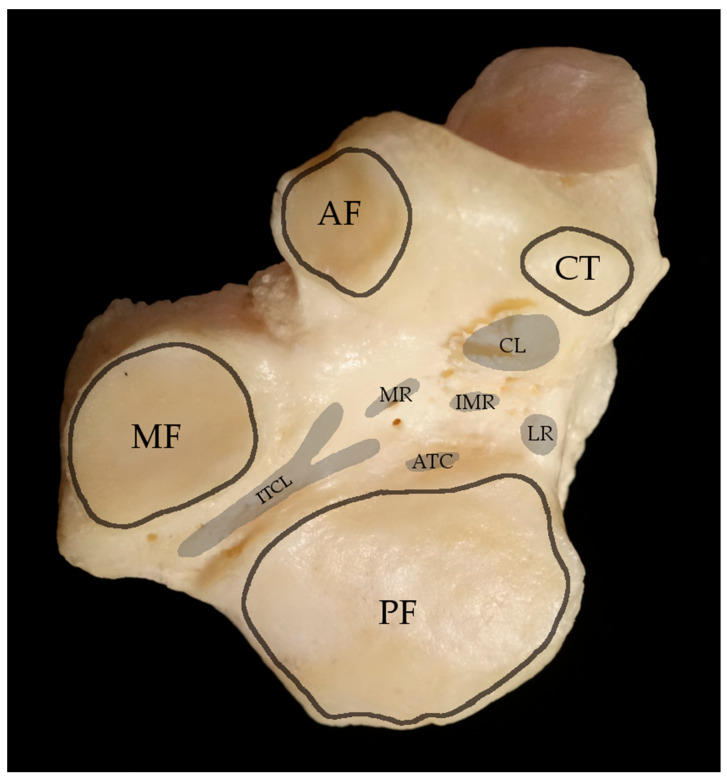
**Ligament attachments in the tarsal sinus and tarsal canal.** Dorsal view of a right calcaneus. AF = anterior facet, MF = medial facet, PF = posterior facet, CT = crista lateralis. The footprints of the cervical ligament (CL), interosseous talocalcaneal ligament (ITCL), anterior talocalcaneal ligament (ATC) and the roots of the inferior extensor retinaculum are highlighted in grey. MR = medial root, IMR = intermediate root, LR = lateral root.

The cervical ligament (CL), the interosseous talocalcaneal ligament (ITCL), and three roots of the inferior extensor retinaculum (IER) are the main stabilizing structures found in the tarsal sinus and canal. The ligament complex of the tarsal sinus and the talotarsal joint are essential for normal movement and stability of the talotarsal joint (Figure 2) [15,16].

Other stabilizing ligaments include the calcaneofibular ligament (CFL), the anterior talocalcaneal ligament (ATC), and the bifurcate ligament (calcaneonavicular and calcaneocuboid ligament) on the lateral side. Medially, there is the medial collateral ligament complex (deltoid ligament with tibionavicular, tibiospring, and tibiocalcaneal parts) and the anterior and posterior tibiotalar ligaments. On the plantarmedial aspect is the spring ligament complex (superomedial ligament, medial plantar oblique ligament, and inferior plantar ligament). The posterior facets of the talus and calcaneus are larger than the middle and anterior facets. They are separated from the other two facets by the interosseous calcaneal ligament [14,20].

In the posterior compartment of the subtalar joint, the concave facet of the talus lies on the convex posterior facet of the calcaneus, forming the subtalar joint. The anatomy of the subtalar joint is extremely variable between individuals in terms of facet size and shape [14,15,16].

The sustentaculum tali is a prominent bony structure formed by the middle calcaneal facet and provides a sliding surface for the tibialis posterior, flexor hallucis longus, and flexor digitorum longus tendons at its plantar surface.

### 3.2. Biomechanics

The talotarsal joint, as already mentioned, is subdivided into the talocalcaneonavicular joint and the subtalar joint. It is morphologically a complex multiplanar joint with an oblique axis. The subtalar joint, or talocalcaneal joint, is a saddle-shaped joint and has a convex upward orientation. Its motion has been compared with a mitered hinge [21]. The talocalcaneonavicular joint, the acetabulum pedis, is thus also coupled with the talocalcaneal joint motion. The combined axis of the functional parts of the talotarsal joint shows some variations, with an inclination in the sagittal plane of 46° ± 9° and a medial deviation in the horizontal plane of 23° ± 11° relative to the axis of the foot [22].

The unique composition of the talotarsal joint and the orientation of its facets allow for motion of the included bones in three planes. These movements together then define the range of motion for the talotarsal joint complex as 25–30° of inversion/supination and 5–10° of eversion/pronation [23,24,25].

The described triplane motion in combination with plantarflexion/dorsiflexion of the talocrural joint (ankle joint) is crucial for walking and adjustments to uneven ground. With the subtalar joint everted with the hindfoot in valgus, the transverse tarsal joints are “unlocked”, which allows a smooth transition from heel strike to stance phase. As gait progresses through the stance phase, the hindfoot goes into varus, and the subtalar joint inverts, “locking” the transverse tarsal joints, providing a rigid lever allowing propulsion forward [26].

### 3.3. Etiology

Numerous etiologies of STS have been proposed. In 1960, Brown suggested it was caused by pinching of the herniated synovial membrane in the tarsal sinus [27], and the concept of soft tissue impingement has been described in more recent literature as well [9,28]. Other etiologies include damage to ligaments of the tarsal sinus [29,30], bleeding in the sinus [4,5], arthritis or synovitis of the talotarsal joints [4,5], and chronic inflammation of the fibro-adipose tissue in the sinus [4,30]. Lateral impingement has also been proposed either due to posterior tibial tendon insufficiency causing diminished inversion [31], valgus hindfoot alignment [32,33], or from anatomic variations such as an accessory anterolateral facet [34,35,36] (Table 1).

**Table 1:** Differential diagnoses and possible causes of STS.

**Figure 3:**
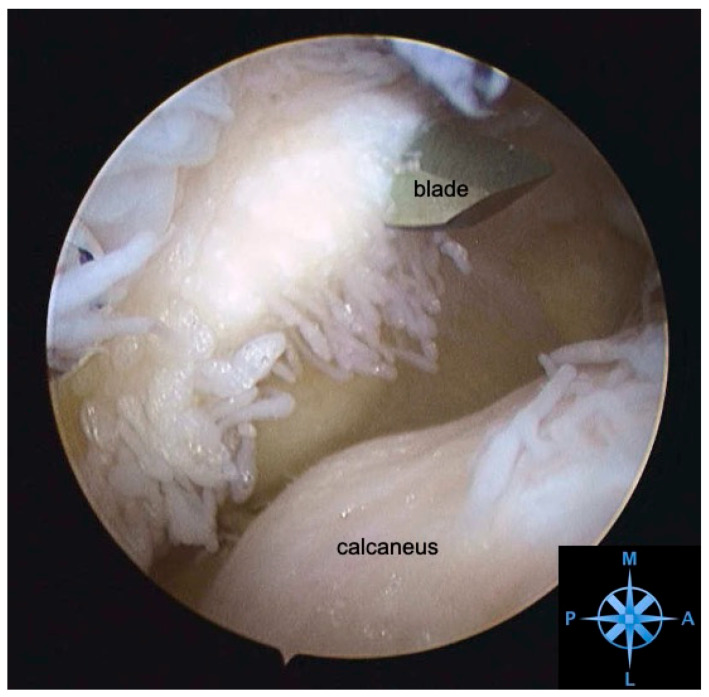
**Arthroscopic view of the posterolateral recess with severe synovitis.** Subtalar arthroscopy of a right hindfoot. View from the anterolateral sinus tarsi portal towards the posterolateral recess with extensive synovitis. A posterolateral portal was created (blade) under direct visualization to debride the recess. M—medial, L—lateral, P—posterior, A—anterior.

**Figure 4:**
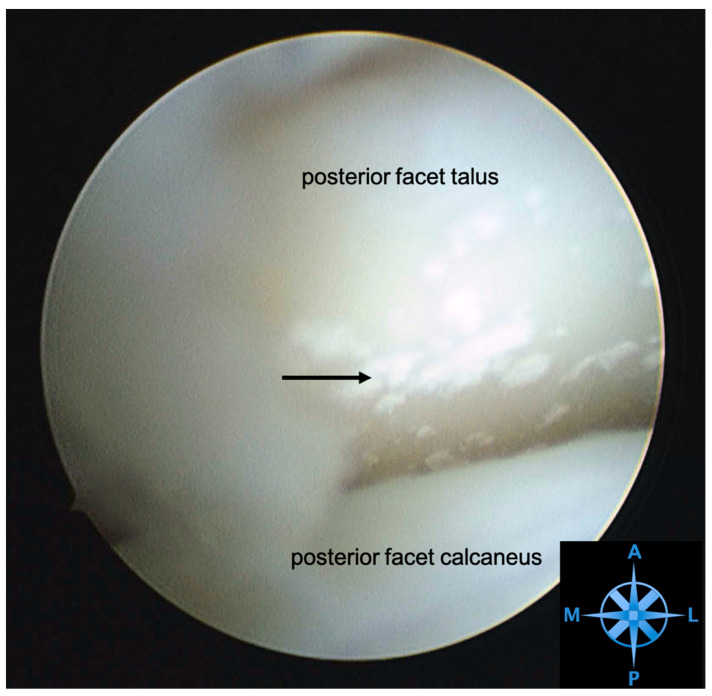
**Arthroscopic view of a posterior facet with chondrocalcinosis.** Subtalar arthroscopy in a right hindfoot. View from the posterolateral portal towards the talocalcaneal joint. Dorsal the posterior facet of the talus shows signs of chondrocalcinosis (arrow). M—medial, L—lateral, P—posterior, A—anterior.

**Figure 5:**
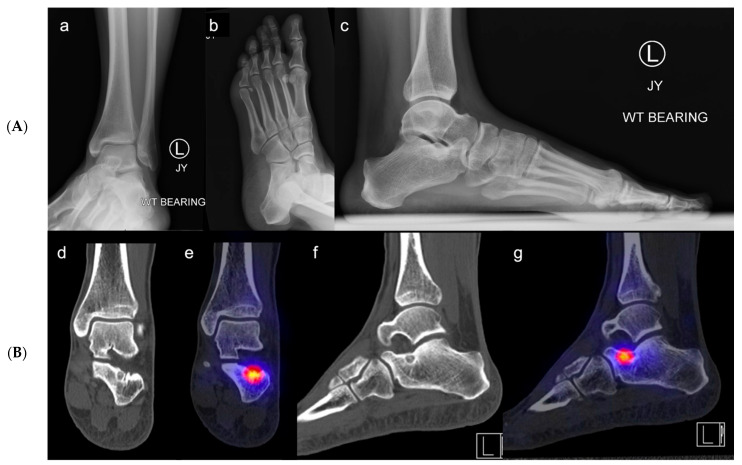
(**A**,**B**). **Osteoid osteoma of the calcaneus at the floor of the sinus tarsi.** Case of a 26-year-old female patient with left sinus tarsi pain. (**a**) ap, (**b**) oblique, (**c**) lateral X-rays (**A**) of the left foot and ankle. On the lateral X-ray view (**c**) there are sclerotic changes in the calcaneus at the area of the calcaneal sulcus. Further imaging with SPECT-CT was performed (**d**–**g**). (**d**,**e**) show coronal views with a cystic and sclerotic intraosseous lesion with a central nidus. (**f**,**g**) show sagittal views with the nidus on the floor of the sinus tarsi. On the SPECT-CT imaging (**B**) (**e**,**g**) there is increased uptake at the site of the lesion. The bony lesion is highly suspicious for osteoid osteoma.

− subtalar joint synovitis (Figure 3)
− synovial cysts
− ankle instability
− subtalar instability
− chronic inflammation and infiltration of fibrotic tissues (post-traumatic arthrofibrosis)
− partial tear of the interosseous talocalcaneal ligament (ITCL) or cervical ligament (CL)
− rheumatoid arthritis
− gouty arthritis
− calcium pyrophosphate (CPP) disease/chondrocalcinosis (Figure 4)
− cartilage injury/osteochondral lesion
− osteochondritis dissecans (OCD) (of the posterior facet)
− loose bodies
− tarsal coalition (talocalcaneal, calcaneonavicular)
− posterior tibial tendon tear/insufficiency
− pes planovalgus/flatfoot/hindfoot valgus
− calcaneofibular impingement
− elongated lateral talar process
− degenerative joint disease/osteoarthritis (subtalar)
− implant related pain (sinus tarsi implants)
− neuroma (deep peroneal nerve)
− accessory ossicles (i.e., symptomatic os peroneum, os subfibulare)
− accessory anterolateral talar facet
− tumor/metastasis (i.e., osteoid osteoma (Figure 5)

Instability of the talocrural and talotarsal joints has also been described in relation to STS [37,38]. The instability can be either mechanical or functional. Freemann et al. [6] hypothesized that functional instability is caused by a proprioceptive deficit resulting from partial nerve damage to sensory nerve endings in the joint. In contrast, mechanical instability is described as an abnormal movement due to ligament rupture, elongation, or bony avulsion. The two most common ligaments involved in subtalar joint instability are the calcaneofibular ligament (CFL) and the interosseous talocalcaneal ligament (ITCL) (Figure 2). A combination of mechanical and functional instability may occur [39]. According to Pisani et al. [7], a so-called “subtalar instability syndrome” may be related to either a loss of active stability due to neuromuscular pathology, often a proprioceptive disorder, or to a loss of passive stability due to damage to the ligaments that restrain the talotarsal joint. Ankle trauma or sprains can lead to ligamentous injuries in the talotarsal joint complex [40].

Vascular causes have also been considered. Schwarzenbach et al. [18] published that injury could cause posttraumatic fibrotic changes in the wall and surrounding tissue of the veins in the tarsal sinus, leading to a disturbance of venous outflow. This, therefore, would create an increase in intrasinusal pressure, leading to STS.

Akiyama et al. [41] described in their study that STS may also result from disorders of nociception and proprioception in the foot. Commonly, branches from the deep peroneal nerve innervate the tarsal sinus, but also sural nerve innervation may be involved [42]. Rein et al. [43] showed that free nerve endings are the dominant mechanoreceptor type in the ankle ligaments, followed by Ruffini endings. The subsequent predominance of Ruffini endings serves to maintain control of joint position and kinesthesia. Akiyama et al. [41] found abundant free nerve endings and three types of mechanoreceptors (Pacinian corpuscles, Golgi-like endings, and Ruffini endings) in the sinus tarsi, indicating that the sinus is not only a joint space but a source of nociceptive and proprioceptive information on the movement of the foot and ankle, adding to the hypothesis that they may play an important role in the pathophysiology of STS.

### 3.4. Clinical Evaluation and Diagnosis

#### 3.4.1. History and Physical Examination

Given the multiple causes of sinus tarsi pain, a detailed medical history is required, including a thorough pain history. This should include questions about symptom duration and location, when pain occurs, ability to participate in sports, associated instability, history of previous trauma, and prior treatment or operations on the foot or ankle. A complete medical history to elicit other causes of hindfoot pain, such as infection, arthritis, and gout, should be obtained. All patients with STS symptoms report tenderness or pain in the sinus tarsi area. Distinguishing between ankle and subtalar instability is difficult because the clinical symptoms of both are very similar [25]. In the latter, most patients complain of a feeling of “giving away” or “rolling over”. Additional symptoms of instability include problems during walking on uneven ground, recurrent pain, and swelling.

A focused assessment and clinical examination of the foot and ankle, as well as an evaluation of gait, should be performed. Assessment of hindfoot alignment should be noted. Inspection for swelling, warmth, and redness of the hindfoot, as well as a thorough neurovascular exam, are imperative. Palpation of the sinus tarsi is essential, specifically with dynamic eversion to replicate impingement. Clinical measures of talotarsal range of motion can be challenging. Some have suggested that manual exam of isolated subtalar motion is not accurate since several joint complexes are involved during inversion and eversion of the foot and ankle [25]. The key to deciphering the cause of instability is an astute physical examination of the ankle, subtalar, and midfoot stability assessing specifically the ATFL with the anterior drawer of the ankle in 20° of plantar flexion, the CFL with the anterior drawer of the ankle in neutral dorsiflexion and varus tilting, subtalar hyperlaxity with the anterior drawer in 90° as well as varus stressing with the ankle stabilized, and midfoot hyperlaxity should be assessed for excessive inversion and eversion of the midfoot, as well as gait and strength analysis to assess the firing of the peroneals that dynamically stabilize the joint.

To date, there are no described pathognomonic signs or special tests to diagnose STS based on history or physical examination. However, the senior author’s dynamic impingement test is felt to be a strong clinical indicator for sinus tarsi impingement. This test is performed by everting the hindfoot and simultaneously palpating the sinus tarsi. In addition, pain relief after injection of local anesthetic or cortisone into the sinus tarsi is strongly suggestive of STS [1]. Injections can also be used as a therapeutic tool [1,2].

#### 3.4.2. Radiographic Evaluation

Appropriate imaging of patients assumed to have STS is a critical part of determining the treatment algorithm. To evaluate malalignment and deformity, specifically to identify a potentially causative planovalgus foot, standing anteroposterior (AP) and lateral radiographs of the foot and ankle are necessary (Figure 5A). Additional views such as the ankle mortise view, oblique of the foot, and hindfoot alignment view (Saltzman view) can be helpful as well. Bilateral radiographic assessment should always be done for comparison.

As evaluation of the talotarsal joint on conventional radiographs is difficult, other radiographic techniques to visualize the talotarsal joint have been proposed. The Broden view is used to view the posterior facet in a lateral projection. The patient is supine with the leg internally rotated 30° to 45°. The center beam is placed in the lateral malleolus. The x-ray tube is then angled sequentially in 10-degree increments from 10 to 40 degrees cephalad [14,25,44]. An axial calcaneal projection, the Harris–Beath view, can demonstrate the middle and posterior facets of the talotarsal joint, with a tangential projection of the sustentaculum tali [44].

If there is any evidence of a limb length discrepancy or rotational long bone deformity, a standing AP long leg radiograph and rotational CT scan of the legs are recommended, respectively.

Stress radiographs performed by manual traction, or a Telos device, have been described to assess mechanical subtalar joint instability [23,25]. The subtalar joint can be assessed from the lateral radiographic view when an anterior drawer maneuver is completed in neutral dorsiflexion of the ankle. When there is anterior translation of the posterior facet of the calcaneus on the talus of more than 7 mm, subtalar instability is suspected [45]. On the varus stress view, subtalar instability is suspected when a loss of parallelism is noted between the posterior facet of the calcaneus and the talus [1,37]. These stress radiographs are rarely used in clinical practice to diagnose talotarsal joint instability due to the high rate of false-positive examinations [25]. A recent review of available imaging modalities to detect subtalar joint instability concluded that currently used imaging techniques cannot accurately predict subtalar joint instability [38].

#### 3.4.3. Advanced Imaging

Since talotarsal pathology is difficult to identify on X-rays, the use of cross-sectional multiplanar imaging is often recommended to better visualize the complex joint anatomy and improve the sensitivity and specificity of detecting subtalar joint pathology. Computed tomography (CT) imaging is the modality of choice if bony pathology is suspected, including arthritis, hindfoot malalignment, bony impingement due to elongated lateral talar processes [34], bony tarsal coalitions [46], and tumors (i.e., osteoid-osteoma, cysts) [47]. Weight-bearing CT scans may be better able to assess the association between malalignment and impingement, inform the design of offloading orthotics, and help guide appropriate surgical correction. Unfortunately, this modality is not readily available in many institutions [48,49,50].

Single-photon emission computerized tomography (SPECT) has the benefit of marrying the image of a radioisotope bone scan with the cross-sectional imaging of a CT, allowing for a more specific analysis of areas of impingement and increased uptake [51,52] (Figure 5B).

Magnet resonance imaging (MRI) of the hindfoot is commonly performed to evaluate talotarsal joint and soft tissue pathology, including sinus tarsi syndrome [28,53]. MRI can help identify other potential causes of sinus tarsi pain. Signals in soft tissues might show tibialis posterior tendon pathology, CFL integrity, interosseous ligament disruption/hypertrophy, or the presence of scar tissue formation or tumors. The diagnosis of interosseous ligament injury can be challenging due to the variable joint anatomy and the three-dimensional orientation of the ligaments in the sinus, which are seen best on sagittal and coronal views [1]. Increased T2 and decreased T1 signal within the marrow (i.e., marrow edema) of the talus and calcaneus could indicate bone marrow contusions and micro trabecular changes (i.e., fractures or bone bruises) from altered biomechanics/malalignment or trauma [54]. Increased signal intensity on T2/STIR-weighted images might represent subtalar joint synovitis or effusion, while decreased signal intensity on Tl-weighted images would suggest sinus tarsi fibrosis or scar formation [10]. Articular cartilage lesions can also be identified. MRI has high sensitivity but unknown specificity in the evaluation of sinus tarsi pain [53].

Arthrography of the subtalar joint has been reported as a way of diagnosing sinus tarsi syndrome, but there are no clear criteria for pathological findings [4]. Arthrography of the subtalar joint is more frequently done while simultaneously performing a fluoroscopic intra-articular injection of local anesthetic and corticosteroid as a diagnostic and therapeutic modality. CT arthrogram can show more bony detail than plain arthrography and further delineate specific three-dimensional morphology or bony defects [55]. An anterolateral approach to injecting the posterior facet is reported to be technically easier, with less incidence of adjacent tendon sheath puncture or tarsal tunnel neurovascular injury as can occur with a posteromedial approach [56].

#### 3.4.4. Arthroscopy

Subtalar arthroscopy allows for direct visualization of the joint and ligaments for accurate diagnosis and concurrent treatment. Arthroscopic evaluation of the tarsal sinus and the subtalar joint of STS patients can reveal tears of the interosseous ligament, cervical ligament, arthrofibrosis, and degenerative joint disease (Figure 3 and Figure 4). A prospective study evaluated the diagnostic accuracy of MRI compared to arthroscopy in 30 feet with STS. They found that ITCL ruptures were underestimated by MRI compared to subtalar arthroscopy. MRI had a sensitivity of 44% and a specificity of 60% [57]. Arthroscopy is therefore a powerful tool for confirming the absence of causative pathology for STS symptoms [1,10,57].

### 3.5. Treatment

Most authors initially treat STS with non-operative modalities, including sinus tarsi injections with corticosteroids or local anesthetics, activity modification, and physical therapy [4,11,28]. Taillard et al. [4], who reported on STS treatment, described successful non-operative treatment in about two-thirds of patients. Surgical treatment techniques vary. More remote studies describe open sinus tarsi decompression, including removal of the contents of the lateral half of the sinus tarsi, and showed improvement or complete resolution of symptoms in up to 90% of cases [2,4]. Nevertheless, precise descriptions of the surgical technique, including which contents of the tarsal sinus were removed, are lacking. Dellon et al. [9] described open denervation of the terminal branches of the deep peroneal nerve in 13 STS patients. At a minimum of 6 months postoperatively, 10 patients (77%) were completely pain free, wore normal shoes, and had returned to work. Kashuk et al. [58] reported that arthroscopic techniques for decompression of the tarsal sinus were successful, technically easy, and enabled a rapid recovery. More recent data support the idea that subtalar joint arthroscopy is a safe and effective tool for the diagnosis and concurrent treatment of sinus tarsi pain and pathology [10,11,12,59,60,61].

## 4. Discussion

**Figure 6:**
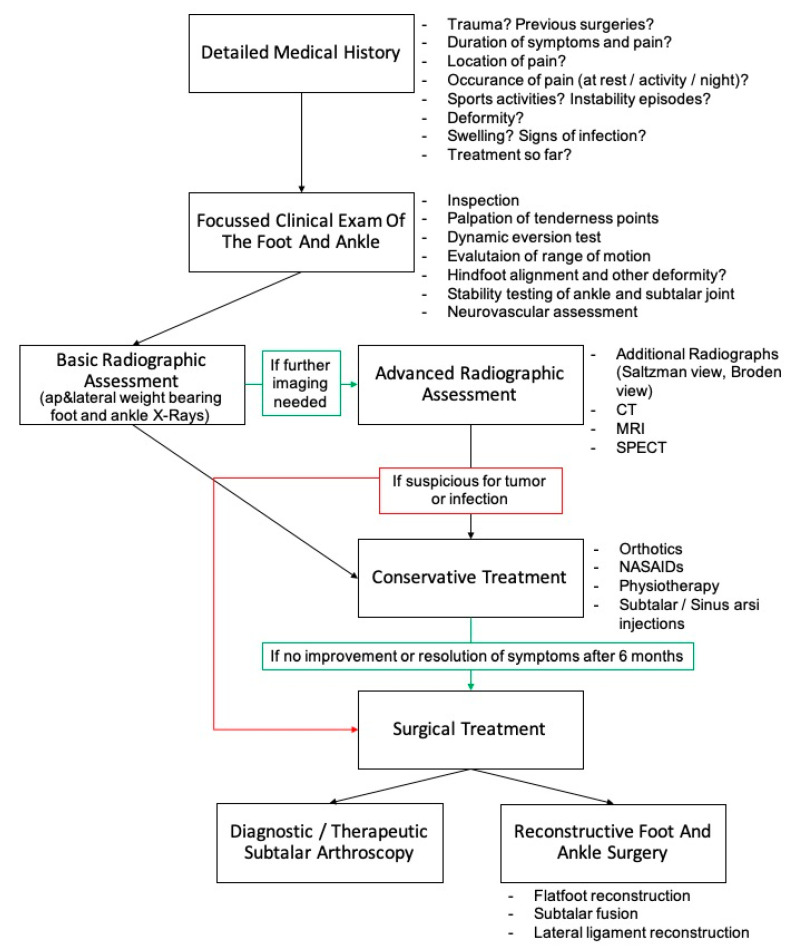
Proposed diagnostic approach to evaluate sinus tarsi pain including treatment.

The literature describes many clear pathologies and causes for pain around the subtalar joint and sinus tarsi. While these are often labeled as STS, the term STS is still frequently used to describe non-specific pain in the sinus tarsi area. In the authors’ opinion, STS will almost always have a causative pathology if one employs an appropriate and detailed history, physical exam, and radiographic or arthroscopic evaluation (Figure 6). The key to successful treatment of sinus tarsi pain, or STS, is to determine a precise etiology that will inform a successful treatment.

If valgus hindfoot alignment or a planovalgus foot are noted, the resulting lateral bony and soft tissue impingement may be causing the sinus tarsi pain. Mechanical offloading the tarsal sinus is needed. Conservative treatment with corrective orthotics, physical therapy, and bracing can be trialed first. If symptoms persist, surgical deformity correction with bony and soft tissue procedures is needed.

If subtalar instability is noted, physiotherapy, including dynamic stabilizer strengthening exercises, proprioception training, and bracing of the hindfoot, should be trialed. If this fails, lateral ligament reconstruction may be necessary [7,21,62]. If there is any concomitant planovalgus alignment, the deformity is also corrected to avoid exacerbating lateral impingement due to suture placement or the development of postsurgical scaring in the sinus tarsi area.

If intraarticular subtalar pathology is not identified on imaging and conservative treatment is not leading to pain relief after a minimum of 6 months, subtalar arthroscopy should be performed [10,21,58,62]. Frey et al. [1] evaluated 14 patients with STS symptoms of unknown cause based on clinical and imaging assessments with subtalar arthroscopy. Ten patients had interosseous ligament tears, two had arthrofibrosis, and two had degenerative joint disease. They also described scar formation, gross hyalinization of the torn ends of ruptured subtalar ligaments, and subsequent impingement of soft tissue into the anterior aspect of the subtalar joint. Frey et al. [1] called this a subtalar impingement lesion (STIL). They therefore questioned the term “sinus tarsi syndrome”, as in all cases, a distinct cause for sinus tarsi pain was found.

In the last decade, subtalar arthroscopy and sinus tarsi endoscopy outcomes have evolved dramatically with improved techniques, increased surgeon experience, and the development of specialized small joint instrumentation. Its use as a diagnostic and therapeutic tool for the evaluation and treatment of sinus tarsi syndrome is considered safe and effective. Compared to open techniques, it is reported to have faster postoperative recovery, decreased postoperative pain, and fewer complications [63]. Frey et al. [1], above, report a success rate of 94% with good to excellent patient-reported outcomes for treating a variety of etiologies. Oloff et al. [10] reported on a series of 29 patients with STS who were arthroscopically managed. Eighty-six percent of patients had a history of previous trauma including 15 patients with previous inversion injuries. The mean return to full activity was, 4 months (range, 2–12 months), and the overall mean postoperative AOFAS score was 85 points (range, 59–100). Lee et al. [11] performed 33 subtalar arthroscopies for STS and found a partial tear of the interosseous talocalcaneal ligament in 88% of cases. Other findings included synovitis (55%), partial tear of the cervical ligament (33%), arthrofibrosis (24%), and soft-tissue impingement (21%). The mean AOFAS ankle-hindfoot score improved from 43.1 points preoperatively to 86.2 points postoperatively. Of the cases, 16 (48%) had an excellent result, 13 (39%) had a good result, and 4 (12%) had a fair result.

Li et al. [12] treated 57 patients with STS combined with chronic lateral ankle instability with arthroscopy and found a partial tear of the interosseous talocalcaneal ligament in 4 patients (7%), arthrofibrosis in 50 patients (88%), synovitis in 46 patients (81%), and cartilage injury in the subtalar joint in 2 patients (4%), who were treated with arthroscopic debridement of the tarsal sinus and consecutive lateral ankle ligament reconstruction or repair. After a mean follow-up of 30 months, the modified AOFAS score increased from 62.5 to 93, the Karlsson score from 57 to 90, and the Tegner score from 1 to 5, respectively.

Since ankle and talotarsal instability often coexist [12] and are difficult to differentiate, a Broström procedure reconstructing the anterior talofibular ligament (ATFL) with additional Gould augmentation by the IER (which also stabilizes the talotarsal joint) is recommended [25]. In severe cases of lateral ankle instability with signs of subtalar involvement, an anatomical reconstruction of the calcaneofibular ligament (CFL) may be added [25]. Regarding the importance of the ITCL as a stabilizer of the subtalar joint complex, the idea of ITCL reconstruction appears reasonable and promising. Miralles-Marrero et al. [64] were the first to describe a repair of the ligaments of the talotarsal joint in 1978. Subsequently, reconstruction of the ITCL using a partial Achilles tendon graft was reported by Kato et al. [65]. They showed satisfactory patient-reported results with good functional results and low rates of complications. Pisani et al. [7] described an open technique for reconstruction using the anterior portion of the peroneus brevis tendon in 47 patients with good results. A modified arthroscopically assisted technique for ITCL reconstruction with a hamstring autograft has been described [66]. More data on ITCL repair or reconstruction are needed.

## 5. Conclusions

In conclusion, sinus tarsi syndrome (STS) is an imprecise term that is used to describe any sinus tarsi area pain. We recommend surgeons drop the term STS in exchange for the causative etiology, when identified, as this will dictate the treatment. Given that the evolution of sinus tarsi and subtalar arthroscopy allows for more detailed and accurate evaluation of sinus tarsi pathology, it can be used as an effective diagnostic tool that will allow concurrent management of pain sources. Whenever there is planovalgus alignment with subsequent sinus tarsi impingement, this should be treated accordingly. Subtalar or ankle joint instability is another differential diagnosis that can be treated successfully to relieve pain and symptoms.
